# Persistent Supercooling of Reproductive Shoots Is Enabled by Structural Ice Barriers Being Active Despite an Intact Xylem Connection

**DOI:** 10.1371/journal.pone.0163160

**Published:** 2016-09-15

**Authors:** Edith Kuprian, Tan D. Tuong, Kristian Pfaller, Johanna Wagner, David P. Livingston, Gilbert Neuner

**Affiliations:** 1 Institute of Botany, University of Innsbruck, Innsbruck, Austria; 2 USDA-ARS and North Carolina State University, Raleigh, NC, United States of America; 3 Division of Histology and Embryology, Innsbruck Medical University, Innsbruck, Austria; Iwate Daigaku, JAPAN

## Abstract

Extracellular ice nucleation usually occurs at mild subzero temperatures in most plants. For persistent supercooling of certain plant parts ice barriers are necessary to prevent the entry of ice from already frozen tissues. The reproductive shoot of *Calluna vulgaris* is able to supercool down to below -22°C throughout all developmental stages (shoot elongation, flowering, fruiting) despite an established xylem conductivity. After localization of the persistent ice barrier between the reproductive and vegetative shoot at the base of the pedicel by infrared differential thermal analysis, the currently unknown structural features of the ice barrier tissue were anatomically analyzed on cross and longitudinal sections. The ice barrier tissue was recognized as a 250 μm long constriction zone at the base of the pedicel that lacked pith tissue and intercellular spaces. Most cell walls in this region were thickened and contained hydrophobic substances (lignin, suberin, and cutin). A few cell walls had what appeared to be thicker cellulose inclusions. In the ice barrier tissue, the area of the xylem was as much as 5.7 times smaller than in vegetative shoots and consisted of tracheids only. The mean number of conducting units in the xylem per cross section was reduced to 3.5% of that in vegetative shoots. Diameter of conducting units and tracheid length were 70% and 60% (respectively) of that in vegetative shoots. From vegetative shoots water transport into the ice barrier must pass pit membranes that are likely impermeable to ice. Pit apertures were about 1.9 μm x 0.7 μm, which was significantly smaller than in the vegetative shoot. The peculiar anatomical features of the xylem at the base of the pedicel suggest that the diameter of pores in pit membranes could be the critical constriction for ice propagation into the persistently supercooled reproductive shoots of *C*. *vulgaris*.

## Introduction

In alpine environments, freezing temperatures during winter usually do not cause severe damage to plants due to sufficient snow cover and seasonal development of frost resistance [1,2]. In contrast, episodic freezing events during the growing period in summer, which are a peculiar feature of the alpine environment [3], are not predictable and impact plants in a fully frost dehardened state. The frequency and severity of these frost events increases significantly with increasing elevation [4], and can expose alpine plants to freezing temperatures that occasionally can exceed the frost resistance of their tissues [5].

Different tissues and organs show significantly different degrees of frost resistance in summer. In a recent review of alpine woody plants, reproductive tissues were the most susceptible to frost (mean LT_10_ = -4.6°C), followed by premature shoots and leaves (-5.0°C), mature leaves (-6.6°C), vegetative buds (-7.3°C), and finally xylem tissue (-10.8°C) [6]. In a survey of frost resistance in seven common species from the European Alps differing in growth form (dwarf shrubs, herbs, cushion plants), altitudinal distribution and phenology (early-, mid-, late-flowering), reproductive shoots were significantly less frost resistant than vegetative shoots (mean difference for LT_50_−4.2 ±2.7K) during all reproductive stages (shoot elongation, anthesis, and fruiting) [4]. In all species tested, except for *Ranunculus glacialis*, reproductive shoots were sensitive to ice during shoot elongation and anthesis (mean LT_50_ around -4°C), i.e. they were immediately frost damaged when ice formed within their tissue. Therefore, in the alpine environment where radiative cooling may cause leaf temperature minima down to -14 (June) to -24°C (July) and -10 to -20°C (July/August) [6], reproductive organs need special adaptations to allow supercooling so that reproductive success is maintained.

Vegetative plant parts usually show little supercooling capacity in nature and ice forms at mild subzero temperatures [7,8]. After ice nucleation, ice can spread into all plant parts colder than 0°C [9,10]. To prevent spreading of ice into reproductive shoots ice barriers are needed.

Alpine cushion plants (e.g. *Saxifraga bryoides*, *S*. *caesia*, *S*. *moschata*, *Silene acaulis*) are able to take advantage of a steep temperature gradient, due to their cushion growth habit, which serves as a thermal ice barrier [7]. When the capacity to supercool is exceeded, ice nucleation can take place in single reproductive shoots, but ice propagation stops inside the plant cushion, where temperature is above 0°C. This thermally blocks ice from entering into the surrounding inflorescences keeping them in a supercooled state and preventing frost damage [7]. The growth habit of woody shrubs is not compact enough to offer sufficient thermal protection. Recently, for three woody alpine species (*Loiseleuria procumbens*, *Empetrum hermaphroditum*, *Calluna vulgaris*) an effective structural ice barrier between vegetative and reproductive shoots was demonstrated [11]. Despite non-lethal ice formation in the vegetative shoot between -4.7°C and -5.8°C, reproductive shoots were able to remain ice-free and supercool to below -22°C.

Reproductive buds of angiosperms that can temporarily supercool during winter have been reported [3,11–18], but the nature of structural ice barriers between vegetative and reproductive shoots is not well understood [19]. Features of ice barrier tissues include a) provascular strands (*Rhododendron spec*. [12]), b) small tightly packed cells with thick walls that are devoid of vacuoles and intercellular spaces (e.g. *Prunus persica*: [20,21], *Vitis spec*.: [22]), c) impregnation of cell walls with phenolic compounds (suberin and lignin *Rhododendron* species: [23]), d) accumulation of unesterified pectins (*Prunus persica*: [24]), and e) regions of dry tissue (*Rhododendron japonicum*: [12], *Prunus persica*: [25]). But in all cases establishment of xylem continuity during regrowth in spring caused a loss of supercooling capacity (*Prunus persica*: [20], *Forsythia*: [13], *Prunus armeniaca*: [26], *Malus sylvatica*: [27], *Rhododendron ferrugineum*: [3]). In contrast, in *L*. *procumbens*, *E*. *hermaphroditum*, and *C*. *vulgaris* structural ice barriers persisted during shoot elongation, anthesis, and fruiting throughout summer, despite the establishment of a continuous vascular connection into the reproductive shoot [11]. However, the structural factors that facilitate supercooling of reproductive shoots of alpine shrubs at extreme temperatures (down to -22°C), even with an established xylem connection, are not known.

Despite a vascular connection, reproductive shoots of *C*. *vulgaris* are able to supercool, on average, down to -10.9°C during anthesis [11]; this suggests the presence of a persistent, structural ice barrier. In this study, the location of this ice barrier between the reproductive and the vegetative shoot was determined by infrared video thermography (IDTA [9], HRIT [28]). Cytological and histological comparisons using light and scanning electron microscopy, provided evidence for the fundamental structural properties that allow blocking the spread of ice into a vascularly connected, actively growing reproductive shoot and, resulting in the frost survival of that shoot by supercooling.

## Material and Methods

### Plant material and collection site

Supercooling of reproductive shoots and the histological and cytological differences between the ice barrier tissue in vegetative and reproductive shoots were investigated in the evergreen woody shrub *Calluna vulgaris* (L.) Hull. The reproductive shoots of *C*. *vulgaris* supercool throughout reproductive development (shoot elongation, anthesis, and fruiting) [11]. *C*. *vulgaris* is the keystone species of Europe’s heathland systems [29,30]. The species is also commonly distributed in the European Alps and usually inhabits south exposed slopes between 700 and 2500 m [31].

For this investigation, branches of *C*. *vulgaris* were collected on the south exposed slope of the summit of Mt. Patscherkofel (2248 m; 47°12’29.7”N, 11°27’42.7”E). In this area, anthesis of *C*. *vulgaris* occurs between July and September, followed by the development of fruits and seeds, and subsequent establishment of overwintering flower buds in late autumn, which sprout in early summer next year (Edith Kuprian, personal observation).

Twenty branches (10–15 cm in length) from at least 10 individuals were collected during anthesis. Branches were put in sealable plastic bags lined with wet paper towels and transported within 1.5 hours to the Institute of Botany in Innsbruck. Samples were then stored in a cold chamber at +5°C for about 1 h until they were further processed.

Field studies were conducted in a landscape protection area where no special permission for collecting plant samples is needed, as long as the plants are not endangered or protected. We confirm that the field studies did not involve endangered or protected species.

### Freezing treatment

For controlled freezing of branches of *C*. *vulgaris*, a commercial chest freezer (GT 2102, Liebherr, Lienz, Austria) was used. Temperature inside the freezing compartment was controlled by LabView 2012, (Software developed by Othmar Buchner) that utilizes user-defined cooling profiles at ±0.2 K. Temperature was controlled by the use of Positive Temperature Coefficient (PTC) heaters (Nimbus B200, DBK Austria, Krems, Austria) against the permanently cooling chest freezer. All freezing protocols were programmed to start at +2°C, which was initially kept for 45 minutes. Temperature was then lowered at 3 K/h, until -18°C was reached. This temperature profile was close to maximum cooling rates that can naturally occur below 0°C in alpine regions [32].

### Infrared-Thermography

Ice nucleation, ice propagation, and supercooling within branches were monitored with a digital infrared camera (T650sc, FLIR Systems, Danderyd, Sweden). The T650sc infrared camera has a thermal resolution of 0.2 mK. We used a close-up lens (magnifying factor: 1.5X, working distance: 46 mm) to obtain a spatial resolution of 25 μm. The infrared camera was connected to a PC through a USB 2.0 port. Infrared images were recorded at an interval of 10 frames per second. Subsequent processing of the images by IDTA [10,33] was conducted with the software FLIR ResearchIR Max (version 4.20.2.74, FLIR Systems, Danderyd, Sweden). Image overlay was done with After Effects (Adobe Systems Inc., San Jose, CA).

Branches of *C*. *vulgaris* bearing flowers were mounted on a small lift table by double-sided adhesive tape. The size of the image field monitored by the camera was 16 mm x 12 mm. Within this region, flowers, flower stalks and the vegetative main stem were arranged in one focal plane. In order to prevent artificial supercooling of the vegetative shoot, ice nucleation in the vegetative shoot was triggered around -3.3°C by application of a droplet of INA (Ice Nucleation Active) bacteria suspension (*Pseudomonas syringae* van Hall 1902) at the cut end of the vegetative shoot. Additionally, six thermocouples were mounted in close proximity to the flowers of *C*. *vulgaris* to record real temperatures. Thermocouple recordings were collected every 10 seconds by a data logger (CR10X, Campbell Scientific, Utah, USA).

### Fixation, Dehydration, Embedding, Cutting, Staining, and Imaging

For cytological, anatomical and histological investigations of the tissues in the flower stalk and vegetative shoot of *C*. *vulgaris*, ten branches out of the overall collection were randomly chosen. These branches were cut into pieces, each consisting of a vegetative shoot (2 cm in length) bearing several flower stalks and flowers. For fixation, these short branch pieces were immersed in Methanol-FAA, a solution of methanol (45%), formaldehyde (10%), glacial acetic acid (5%) and distilled water (40%) [34]. Fixation in M-FAA lasted for 24 h.

Further steps were conducted in the laboratory of David Livingston (USDA-ARS United States Department of Agriculture–Agricultural Research Service, North Carolina State University). For shipping, the fixed short branch pieces were put into ten 2.0 ml safe-lock centrifuge tubes (Eppendorf AG, Hamburg, Germany) filled with M-FAA, avoiding the inclusion of air. Shipping took 4 days. Samples were additionally treated with M-FAA, with an ethanol dehydration series and then embedded in paraffin using a microwave-assisted protocol previously described [34].

Paraffin embedded tissues were sectioned with a rotary microtome (Leica, Model RM2255, Wetzlar, Germany) at 8 μm and triple stained with Safranin, Fast Green, and Orange G [34]. Safranin, Fast Green, and Orange G are stains for lignified, cutinized, and suberized structures, cellulose walls and cytoplasm, and a counterstain for cytoplasmic content, respectively [35]. Sections were photographed with a Canon Rebel T3i (Canon Inc, Taipai, Taiwan) mounted on a Nikon Eclipse 50i light microscope (Nikon Corporation, Tokyo, Japan) at 100X magnification. A 10 mm, 100 part photo-reticle was used to calibrate measurements in all images.

### Quantitative analyzation of cytological and histological parameters

**Fig 1:**
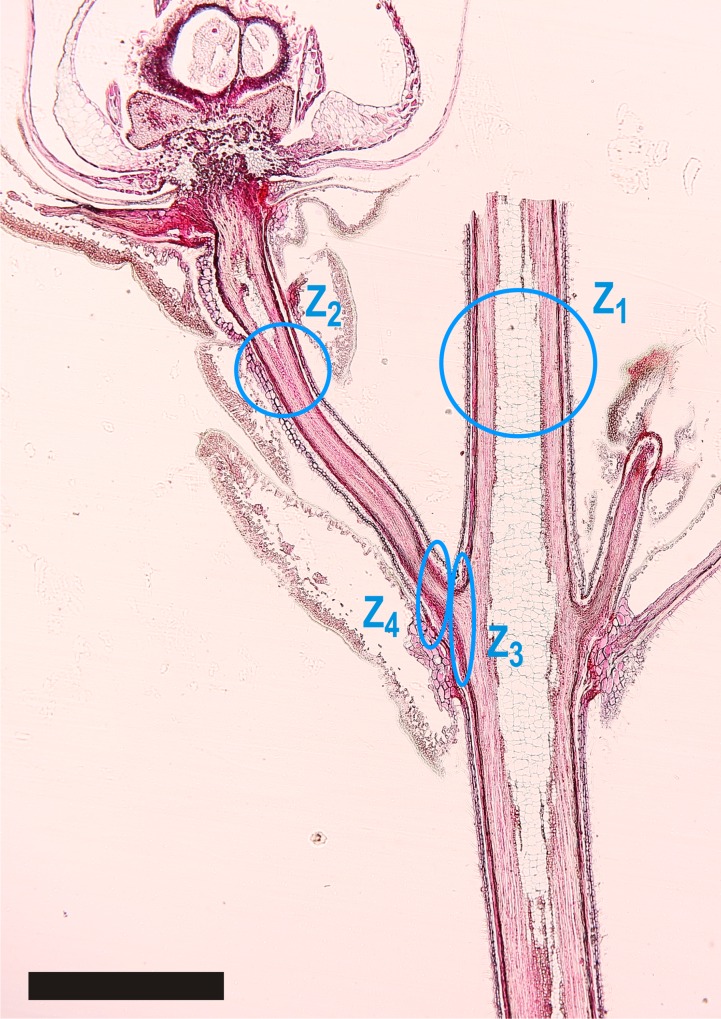
Longitudinal section through a vegetative shoot and an attached reproductive shoot of *C*. *vulgaris* during anthesis. Four different zones were anatomically analyzed in detail (blue circles): (Z_1_) vegetative shoot, (Z_2_) upper part of the pedicel, (Z_3_) transition zone between vegetative shoot and pedicel, and (Z_4_) basal part of the pedicel. The ice barrier tissue refers to both, zone 3 and 4. Black bar = 1 mm.

For quantitative analysis and comparison of cytological, anatomical and histological parameters, four different areas of interest on the shoots of *C*. *vulgaris* (Fig 1) were selected based on the localization of the ice barrier tissue via IDTA: vegetative shoot (Z_1_), upper reproductive shoot (Z_2_), transition zone between vegetative and reproductive shoot (Z_3_), and lower reproductive shoot (Z_4_). Zones 3 and 4 correspond to the tentative ice barrier tissue. Measurements were done on cross and longitudinal sections from 5 individuals.

### Anatomical characterization

The external diameter of the whole shoot (d_e_), diameter of the vascular tissue (xylem) in the shoot (d_x_) and diameter of the pith tissue (d_p_) were quantitatively assessed in all images using Optimas 6.5 (Media Cybernetics, L.P. Optimas, Rockville, MD). The conducting xylem units (tracheae and tracheids; for terminology see [36]) were differentiated by the following characteristics: In cross sections, tracheae and adjacent tracheids were distinguished due to the larger diameter of the tracheae. In longitudinal sections, the tapered ends of tracheids clearly distinguishes them from tracheae and tracheids are longer than tracheae elements [37,38].

Within a 0.00316 mm^2^ area of xylem the number of tracheae and tracheids (n_tt_), the outer diameter (d_tt_), and the cell wall thickness of those xylem elements (t_cw_) were measured. From these values, the ratio of cell wall thickness to cell diameter (r = t_cw_/d_tt_) was calculated. Additionally, the whole xylem area in a cross section (A_x_) was determined by using d_x_ and assuming a circular shape of the vascular tissue in a cross section. If pith tissue was present, the area of the pith tissue was subtracted from the xylem area. By knowing A_x_ and n_tt_ the total number of conducting xylem units in the cross sectional area of xylem (n) was calculated. There were only tracheids found in the transition zone (Z_3_) and the basal part of the pedicel (Z_4_), whose lengths (l_tr_) were measured.

### Scanning electron microscope (SEM) Imaging

Longitudinal sections of *C*. *vulgaris* were dehydrated in a 10, 20, 30, 40, 50, 60% ethanol series for one day at each concentration and then four days in 70% ethanol. After critical point dehydration using Formaldehyd-dimethyl-acetal (FDA), the longitudinal sections were mounted with double-sided adhesive tape containing carbon on an aluminium stub, and sputtered with Au/Pd (80/20; layer thickness 250–300 nm). Examination was done with a Field-Emission-Scanning-Electron-Microscope (DSM 982, Gemini, Zeiss, Oberkochen, Germany). Mean diameter of the slit-shaped pits was calculated based on eight and twelve images taken in the vegetative (Z_1_) and ice barrier zone (Z_3_/Z_4_), respectively.

### Statistical Analysis

Differences in the mean values of ice nucleation temperature between the vegetative and the reproductive shoot, as well as differences in the mean values of cytological, anatomical, and histological parameters between the different areas of interest (Z_1-4_) of the shoots of *C*. *vulgaris* were tested with a one-way analysis of variance (ANOVA) and a subsequent Duncan post hoc test, homoscedasticity provided. In case of a negative Levene test the Mann-Whitney-U-test was used as a non-parametric post hoc test (IBM SPSS Statistics for Windows, Version 21.0. Armonk, NY). All analyses were conducted at a significance level of p ≤ 0.05.

## Results

### Localization of the ice barrier by IDTA

**Fig 2:**
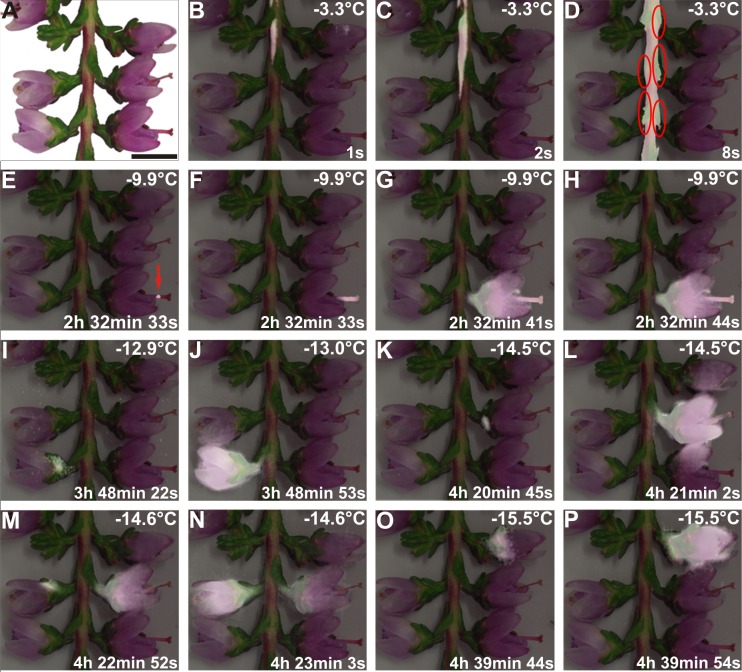
Location of the ice barrier tissue by IR-Thermography. (A) Digital image of a vegetative shoot bearing five reproductive shoots of *C*. *vulgaris* prior to the freezing treatment and (B-P) sequence of images of an overlay of the digital image and of IDTA images during controlled freezing. Heat given off by freezing is white in comparison to the background. Ice nucleated at -3.3°C in an INA bacteria suspension droplet (not in the picture) that had been placed at the cut end of the vegetative shoot and passed immediately into the vegetative shoot (B-D). The structural ice barrier is clearly detectable at the base of the pedicel (D, red circles). All flowers remained supercooled and froze at lower temperatures between -9.9°C and -15.5°C, about 2.5 h and 4.7 h later than the initial ice nucleation in the vegetative shoot. In one flower the initial ice nucleation event was detected in the style (E, red arrow), and ice propagated from there into all other reproductive organs (F-H). The other four flowers showed initial ice nucleation in the region of the calyx, base of the petals or the gynoeceum (I, K, M, O). After nucleation in the flowers ice propagated downwards and stopped at the base of the pedicels (I-P). Actual temperatures are indicated in the top right corner of each image. The time span (in hours, minutes, and seconds) after initial ice nucleation in the vegetative shoot is indicated at the bottom right corner of each image.

Reproductive shoots of *C*. *vulgaris* were able to supercool (Fig 2). To avoid supercooling in the vegetative shoot, a droplet of INA bacteria (*Pseudomonas syringae* van Hall 1902) suspension was positioned at the cut end of the vegetative stem. After ice nucleation in the droplet, ice propagated immediately into the vegetative shoot at -3.3°C (high temperature freezing exotherm, HTE). The ice did not advance into the pedicel, but remained restricted to the main stem suggesting that the ice barrier is localized at the base of the pedicel (Fig 2D). All five reproductive shoots within the recording window of the camera supercooled and froze independently from the vegetative shoot at individually different and significantly lower temperatures between -9.9°C and -15.5°C (low temperature freezing exotherms, LTEs; Fig 2E–2P). Under our experimental conditions, the reproductive shoots remained supercooled between 2.5 and 4.7 h and froze at -9.9, -12.9, -14.5, -14.6, and -15.5°C. Ice nucleation occurred in one flower in the style, but in all others it occurred in the area of the calyx, at the base of the petals or in the gynoeceum. From there, ice spread rapidly throughout all reproductive organs and finally downwards towards the putative ice barrier at the base of the pedicel and the already frozen vegetative shoot. Freezing patterns during both freezing events, HTE and LTE, suggest that the ice barrier is located at the base of the pedicel.

### Anatomy and histology of the ice barrier

The ice barrier tissue appeared to be a constriction zone at the base of the pedicel in the longitudinal section through both the pedicel and the vegetative shoot of *C*. *vulgaris* (see Fig 1). This zone coincides with the region where ice formation stopped at HTE and LTEs in IDTA. The anatomical characteristics in this zone (Z_3_ and Z_4_) were compared to that of the vegetative shoot (Z_1_) and the upper part of the pedicel, where ice can propagate without constraint (Z_2_). In the ice barrier region, two zones were selected for detailed analysis: the transition zone between the vegetative shoot and the base of the pedicel (Z_3_) and the basal part of the pedicel itself (Z_4_).

**Fig 3:**
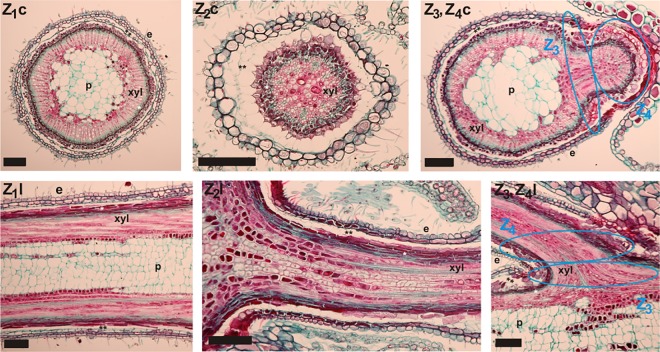
**Images of cross (c) and longitudinal (l) sections of the vegetative shoot (Z1), of the upper part of the pedicel (Z2), of the transition zone between vegetative and reproductive shoot and basal part of the reproductive shoot (Z3, Z4) of C. vulgaris after triple staining with Safranin, Orange G, and Fast Green.** Pith (p), xylem (xyl), phloem (single white asterisk), parenchym (double black asterisk), and epidermis (e) tissue are indicated in the sections. Horizontal black bar = 0.1 mm.

**Fig 4:**
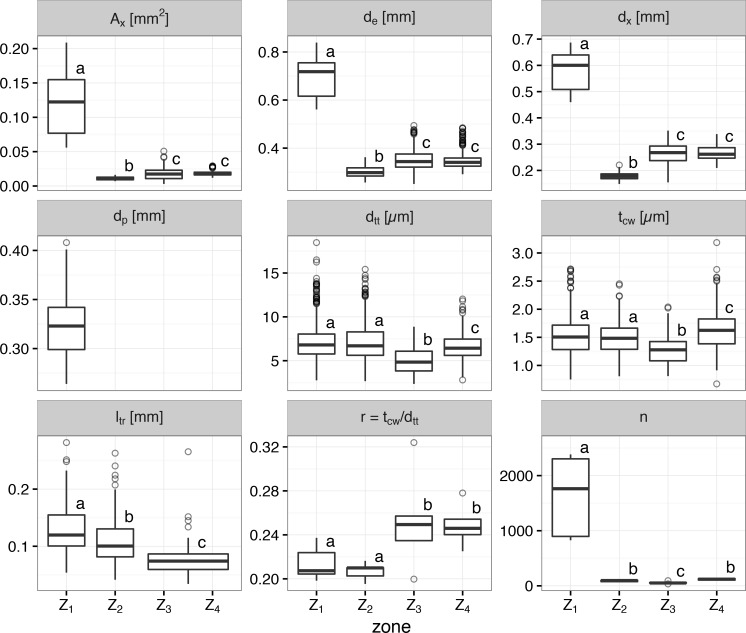
**Anatomical parameters measured on cross and longitudinal sections of the vegetative shoot (Z**_**1**_**), the upper part of the pedicel (Z**_**2**_**), the transition zone between the vegetative shoot and the pedicel (Z**_**3**_**), and the basal part of the pedicel (Z**_**4**_**) in *C*. *vulgaris* (see Fig 1).** Total xylem area in a cross section (A_x_), external diameter (d_e_), diameter of the vascular tissue (d_x_), diameter of the pith tissue (d_p_), diameter of conducting xylem units (depending on zone: tracheae and tracheids or only tracheids; d_tt_), cell wall thickness of conducting xylem units (t_cw_), length of tracheids (l_tr_), ratio of cell wall thickness to cell diameter (r = t_cw_/d_tt_), and number of vessels in a cross section (n), given in mean values ± SE. The box plots indicate the median (= second quartile; line inside the box) and extend from the first to the third quartile. The whiskers show at most the 1.5 fold interquartile range. Outliers are shown as black dots. Within a single parameter, lower case letters indicate significant differences among the mean values of the four zones.

Cross and longitudinal sections from all zones were analyzed (Fig 3). Z_2_, Z_3,_ and Z_4_, which lack pith tissue and intercellular spaces, seemed to be narrower compared to Z_1_ and to contain more compact tissue (Fig 3). In Z_3_ and Z_4_ distinct differences in anatomical, histological and cytological features were detected (Fig 4). The pedicel had a significantly lower external diameter (300–354 μm), approximately half that of the vegetative shoot. Generally, in Z_3_ and Z_4_ pith tissue and tracheae were absent. In Z_2_, some individual pith cells could be seen just beneath the receptacle. In the vegetative shoot, pith tissue had an average diameter (d_p_) of 324 μm, which resembled that of the external diameter in Z_2_ and Z_4_ of the pedicel. Pith cell walls were positively stained with Fast Green (see Fig 3: Z_1_c, Z_1_l). Most cell walls (not only those of tracheids) in the ice barrier region, were positively stained with Safranin, indicating that the cell walls contained hydrophobic substances such as lignin or suberin [35], but only a few cell walls stained green that would suggest increased cellulose inclusions.

Some tracheae and tracheid cell walls stained green and formed several clusters that were evenly distributed in the xylem area of cross sections in Z_1_ and Z_2_. Cell walls of tracheae and tracheids in these zones stained reddish, indicating lignin, cutin, or suberin inclusions [35]. The area of the xylem (A_x_) was 6 to 11 times smaller in the pedicel (Z_2_) as compared to the vegetative shoot. It was smallest in Z_2_, followed by Z_4_ and Z_3_ (Fig 4). Interestingly, in the upper part of the pedicel (Z_2_) d_e_, d_x_ and A_x_ were significantly smaller than in the ice barrier region (Z_3-4_). However, in Z_3_ and Z_4_ only tracheids were found and in the xylem of Z_3_ the length of tracheids was significantly shorter than in all other zones (Z_1, 2 and 4_) (Fig 4). The mean number of tracheids per cross section was lowest in Z_3_ (57.4), followed by approximately twice as many in Z_2_ (93.2) and Z_4_ (117.8) with the highest number in the vegetative shoot (1635.4). In Z_3_, the diameter of tracheids (d_tt_) was smallest with 4.983 μm, when compared to all other zones (Z_1_: 7.123 μm, Z_2_: 7.175 μm and Z_4_: 6.601 μm). Notably, the tracheid length (l_tr_) in Z_3_ and Z_4_ was significantly shorter than in all other zones (Z_1_ and Z_2_). The average length of individual tracheids (l_tr_) in Z_3_ and Z_4_ was 78.2 μm, which was significantly shorter than in the upper pedicel (110.4 μm) and the vegetative shoot (132.5 μm). In total, the ice barrier tissue in Z_3_ and Z_4_ extended about 250 μm in length. Finally, despite the lowest cell wall thickness measured in Z_3_, the ratio of cell wall thickness to diameter (t_cw_/d_tt_) was significantly higher in Z_3_ than in Z_2_ and Z_1_, but not in Z_4_. These data indicate that the xylem architecture was significantly different in Z_3_ suggesting that the movement of ice could have been significantly reduced as it is forced to pass pit membranes in Z_3_ and Z_4_.

### Measurement of pit pore size by SEM

**Fig 5:**
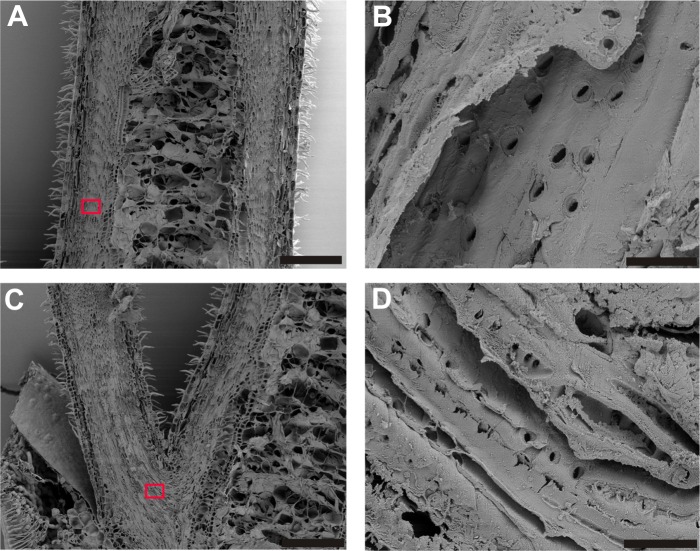
**SEM Images of a longitudinal section of the vegetative shoot (A, B) and the ice barrier zone (C, D) in *C*. *vulgaris*.** Magnifications of the areas indicated by red rectangles (A, C) are shown on the right (B, D). In A and C: black bar = 200 μm, in B and D: black bar = 8 μm.

Slit-shaped pits were found in both the vegetative shoot (Z_1_, Fig 5A and 5B) and in the ice barrier zone (Z_3_/Z_4_, Fig 5C and 5D). Pit apertures in the vegetative tissues were about 2.2 μm long and 0.7 μm wide, whereas in the ice barrier tissue the pit pores were about 1.9 μm x 0.7 μm, which was significantly smaller than in the vegetative part.

## Discussion

### Histological features of the ice barrier

#### Comparison with temporary ice barriers

Thermal observations indicated that ice formation in *C*. *vulgaris* was restricted to the base of the pedicel (Fig 2), where an apparent constriction exists. The external diameter of the pedicel as well as the cross sectional area of the xylem was smaller, no tracheae were found, and the absolute number of tracheids was significantly reduced in this zone. Additionally, tightly packed comparatively small cells with relatively thickened suberized and lignified cell walls, an apparent lack of intercellular spaces, and an increased ratio of cell wall thickness to cell diameter tracheids were found in this zone. These general features of a putative ice barrier have also been reported for temporary structural ice barriers observed in supercooling overwintering reproductive buds [3,11–18,21–23,39]. Additionally, in overwintering buds of angiosperms, the existence of dry tissue regions that were created by withdrawal of water to ice nucleation sites in the bud scales have been suggested (*P*. *persica*: [25], *R*. *japonicum*: [12]). In contrast to our report, these ice barriers are temporary structural ice barriers because the establishment of xylem conductivity during bud break in spring results in a complete breakdown of supercooling (*Prunus* species: [20], *Forsythia* flowers: [13], *Prunus armeniaca*: [26], *Vitis vinifera*: [22], *Malus domestica*: [27], *Rhododendron ferrugineum*: [3]). The undeveloped xylem connection into the meristem is a prerequisite for the functioning of these temporary ice barriers.

#### Xylem features of the persistent ice barrier

The ice barrier in the pedicel of *C*. *vulgaris* functions throughout the year despite the apparent permeability of the xylem to water. This is likely the result of a peculiar xylem structure at the base of the pedicel, which is significantly altered in comparison to the ice penetrable vegetative and reproductive shoots. The xylem of *C*. *vulgaris* is diffuse-porous with mostly isolated vessels of even size, where fiber tracheids make up the main cellular component [40]. In the ice barrier tissue only tracheids were present and due to a reduction of the cross sectional area of the xylem overall a fewer total number of conducting xylem units (-92.8 to -96.5%) is present in the barrier tissue. Additionally, the tracheids in this zone are narrower (-7.3 to -30.0%) and shorter (-41%) with an increased ratio of cell wall thickness to cell diameter (+16.2 to +16.4%) as compared to the vegetative shoot. Very narrow xylem elements (< 20μm) are characteristic of plants in the arctic and alpine zone and are typically observed in small plants [41].

In this study the tracheid length (78.2 μm) was shorter than the length of the ice barrier zone (250 μm), which suggests that ice propagation from the vegetative shoot into the reproductive shoot presumably is restricted by bordered pits. Although pit pore diameter was found to be reduced in the ice barrier zone, forming a constriction, the pit pore diameter is likely still too large to block ice penetration. Hence, it is suggested that the pit membrane could be the key structural element that allows water movement but inhibits ice propagation.

### Cell wall pore size and ice permeability

A critically small pore diameter in the pit membranes may be the key feature of the ice barrier. Water in small pores can remain unfrozen despite direct contact with ice [42]. Theoretically, water could supercool down to -18°C, as observed in some flowers of *C*. *vulgaris*, if cell wall pores in the ice barrier were smaller than approximately 5.2 nm [43]. Ice propagation via cell wall pores would therefore be limited by pore diameter, which reduces the melting point of a liquid in microcapillaries, when the diameter is less than 73 nm [43]. The effect of small pores becomes more apparent at a pore diameter less than 7 nm, which causes a melting point depression of about 10 K [43]. We presume that in the ice barrier tissue of *C*. *vulgaris* a reduced diameter of pores in pit membranes allowed blockage of ice despite unhindered xylem water conductivity. Hacker and Neuner [10] suggested that pits likely restrict ice propagation because highest ice propagation rates were observed in water filled protoxylem lacunae (*Poa alpina*, *Juncus trifidus*), which lack inter-vessel pit membranes. Other observations on ice propagation rates in the xylem e.g. of *Taxus baccata* also reveal that the penetration of ice is restricted when pit membranes must be crossed, i.e. into lateral stems or needles [9]. Generally, lateral ice propagation in the xylem was slower than longitudinal because this usually requires passage through pit pores [9,44]. Further investigations on the diameter of pores in pit membranes in different parts of *C*. *vulgaris* need to be made to get more information about the actual ability of these pores to stop the spreading of ice.

Pectin influences the porosity of the cell wall by regulating the pore size diameter [45]. Non-lignified primary cell walls have pore diameters of between 5–10 nm, pectins restrict the diameter to below 6 nm, which suggests a potential for melting point depression [46]. Accumulation of pectins in intercellular spaces and the middle lamellae of cell walls in the subepidermal layer of the bud scales and in the tissue subtending the floral primordium in the buds of *Prunus persica* has been shown to aid temporary supercooling during winter [24]. Interestingly, enhanced pectin synthesis also appears to play an important role during cold acclimation in non-supercooling tissues [47–51] and in suspension cultures of grape cells [46]. During cold acclimation in apple an increase of cell wall strength due to pectin synthesis and a decrease of pore-size of cell walls from 3.5 to 2.2 nm was reported [46]. Further studies on the role of pectins in the supercooling ability of reproductive shoots of *C*. *vulgaris* need to be made.

### Role of ice nucleators in supercooling

Results obtained by IDTA suggest that the reproductive shoots freeze, not because of a break-down of the ice barrier and ice entrance from the vegetative shoot, but due to separate ice nucleation events somewhere in the reproductive tissue. Hence, the absence of extrinsic and intrinsic nucleation must be accounted for to fully explain the supercooling observed in reproductive tissues (see Fig 2). Extrinsic ice nucleation is commonly prevented by plant cuticles (*Rhododendron* species: [44]; *Vaccinium macrocarpon*: [52]; *Poa alpina* Hacker and Neuner, unpublished), which are hydrophobic barriers to ice propagation. In fact, coating plants with a hydrophobic particle film can increase supercooling further [53]. We suggest that the surface properties of the reproductive shoots of *C*. *vulgaris* must efficiently prevent extrinsic nucleation.

Deep supercooling is usually accomplished by maintaining symplastic water in an unfrozen condition [54]. However, the supercooled reproductive shoots of *C*. *vulgaris* and also of other woody dwarf shrubs like *Loiseleuria procumbens* and *Empetrum hermaphroditum* contain a significant amount of apoplastic water in the xylem that must also be kept in the supercooled state [11]. We suggest that persistent supercooling in reproductive shoots of *C*. *vulgaris* was achieved by the absence of intrinsic ice nucleators and/or an as yet unidentified anti-ice nucleating factor in the apoplast and symplast.

Conclusion: Supercooling in reproductive tissues of *Calluna* appears to be primarily due to a barrier between vegetative and reproductive tissues. This barrier is able to restrict ice propagation into the reproductive shoot due to several distinctive anatomical features. In this region, tracheids are the only water conducting elements present, histological staining suggests that cell walls contain hydrophobic substances, and there are fewer tracheids that are, shorter and narrower, and this tissue lacks intercellulars. Additionally, cell walls in this region are thicker and water is forced to pass pit membranes that are assumably impermeable to ice. While as yet unknown nucleating factors are undoubtedly also important, this study provides a possible explanation to the arguably universal ability of certain plant tissues to supercool and avoid freeze damage.
